# Self-Healing, Remoldable, and Conductive Starch-Based Dual Reversible Cross-Linking Hydrogels for Strain Sensors

**DOI:** 10.1021/acsami.5c05168

**Published:** 2025-06-22

**Authors:** Kai Lu, Xiaolong He, Dian Burhani, Jintao Hu, Petra Rudolf, Dina Maniar, Rudy Folkersma, Vincent S.D. Voet, Katja Loos

**Affiliations:** a Macromolecular Chemistry and New Polymeric Materials, Zernike Institute for Advanced Materials, 3647 University of Groningen, Nijenborgh 3, Groningen 9747AG, The Netherlands; b Circular Plastics, Academy Tech & Design, 84808 NHL Stenden University of Applied Sciences, Van Schaikweg 94, Emmen 7811KL, The Netherlands; c Surfaces and Thin Films, Zernike Institute for Advanced Materials, 3647 University of Groningen, Nijenborgh 3, Groningen 9747AG, The Netherlands; d Research Center for Biomass and Bioproduct, National Research and Innovation Agency, KST Soekarno, Jl. Raya KM.46, Cibinong 16911, Indonesia

**Keywords:** starch, dual cross-linked network, hydrogel, cellulose nanocrystals, self-healing, wearable sensors

## Abstract

Polysaccharide-based hydrogels have been utilized as flexible strain sensors because of their renewability, biocompatibility, and biodegradability. However, their widespread application is hindered by the complexity of their manufacturing processes and the inevitable degradation of their mechanical properties with repeated use. The introduction of reversible bond chemistry offers the potential to impart self-healing properties to hydrogels, extending their functional lifespan. In this study, we prepared a starch-based conductive hydrogel (starch/poly­(vinyl alcohol) (PVA)/cellulose nanocrystals (CNCs)) via a straightforward method using borax as a cross-linking agent. The hydrogel demonstrated improved strength and self-healing property because of the addition of CNCs, which formed dual reversible cross-links with starch and PVA via hydrogen and borate ester bonds. Additionally, the sodium ions (Na^+^) and borate ions (B­(OH)_4_^–^) within the network enhanced the electrical conductivity and strain sensitivity of the hydrogel. The resulting hydrogel demonstrated potential for application as a wearable sensor capable of monitoring a range of human movements, sensing handwriting, and enabling Morse code communication. Notably, the hydrogel could be easily remolded at room temperature after being sectioned, highlighting its practical applicability. This work expands the scope of the use of starch-based hydrogels in sustainable wearable sensor technologies.

## Graphical abstract

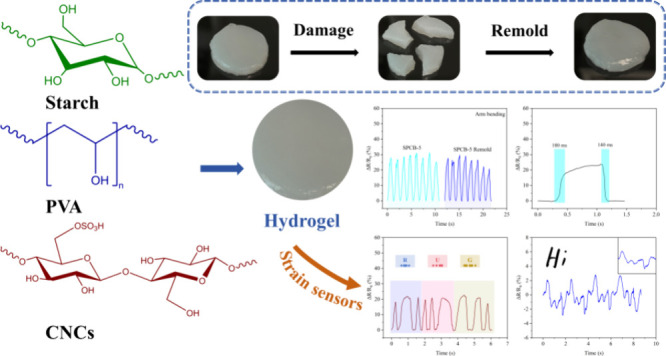

## 1 Introduction

During the past few years, wearable and flexible strain sensors are commonly used in human motion and health monitoring due to their ability to convert mechanical deformation into electrical signals.^1–5^ Hydrogels are able to absorb and hold water inside their networks without disintegrating due to their three-dimensional hydrophilic structures.^6^ They feature good flexibility, stretchability, and sensitivity, making them excellent active materials for flexible strain sensors.^7,8^ However, hydrogels are mainly derived from petroleum-based polymers; the greenhouse gas emissions produced throughout their lifecycle have a significant environmental impact, and when waste, these hydrogels may cause severe environmental pollution.^9,10^ Environmentally friendly sensors arising from polysaccharide hydrogels, including alginate, cellulose, and starch, have gained great interest because of their renewability, biocompatibility, and biodegradability.^11,12^

Starches are among the most abundant polysaccharides in nature and have attracted increasing research interest since their cost is low, and they are renewable and biodegradable.^13,14^ Starch mainly consists of amylose and amylopectin; amylose is a linear polymer, whereas amylopectin is a branched polymer.^15–18^ The abundant hydroxyl groups on starch chains make them suitable to prepare hydrogels via chemically or physically cross-linking.^19^ However, the prepared hydrogels typically have poor mechanical properties and brittleness due to their single cross-linked structure and rigid molecular chains.^20^

The incorporation of synthetic polymers into starch hydrogels to form hybrid hydrogels effectively enhances their mechanical properties. Poly­(vinyl alcohol) (PVA) contains hydroxyl groups in repeating unit that favor network formation through hydrogen bonds or chemical bonds.^21^ PVA is hence suitable for increasing the strength of starch-based hydrogels.^22,23^ Incorporating nanoparticles, including metal nanoparticles and cellulose nanoparticles, is another strategy to reinforcing hydrogels.^24,25^ Interesting nanoparticles are cellulose nanocrystals (CNCs), and they are widely used as polymer reinforcing fillers because of their robust strength, biodegradability, and biocompatibility.^26,27^ Thus, the incorporation of PVA and CNCs into starch-based hydrogels represents an effective strategy for improving their mechanical properties and stability. However, repeated use inevitably leads to mechanical damage in these hydrogels, which poses a challenge to their durability and restricts their widespread application.^28^ Therefore, to increase their durability and reliability, it is important to develop hydrogels that simultaneously exhibit good mechanical properties and self-heal property.

Currently, the design of self-healing hydrogels focuses primarily on incorporating dynamic covalent or noncovalent interactions. Dynamic covalent interactions employed such as Diels–Alder reactions, as well as borate ester bonds, imine, and disulfide.^29–32^ While noncovalent interactions include hydrophobic interactions, hydrogen bonds, and ionic bonds,^33–36^ borax is an effective cross-linking agent, which can be used to prepare self-healing hydrogels via borate ester bonds between the hydroxyl groups of polyols.^37,38^ Moreover, these hydrogels exhibit conductive ability and strain sensitivity due to sodium ions (Na^+^) as well as borate ions (B­(OH)_4_^–^) within the network.^39^ Lu et al. developed hydrogels with increased stiffness and self-healing property, which were attributed to the borate ester bonds formed between borax and the hydroxyl groups of PVA/microfibrillated cellulose (MFC).^21^ Wang et al. reported a starch-based conductive hydrogel that showed an ultrafast self-healing capability and high electrical conductivity. This performance arises from hydrogen bonds together with dynamic borate ester bonds, as well as the abundant Na^+^ and B­(OH)_4_^–^ within the networks. However, the preparation is time-consuming, and the reuse of hydrogels requires heating and cooling processes.^40^ On the basis of these findings, we hypothesize that incorporating PVA, CNCs, and borate ions into starch-based hydrogels could prepare hydrogels with simultaneously enhanced strength, self-healing capabilities, and improved conductivity.

Herein, we prepared a starch-based conductive hydrogel composed of starch, PVA, and CNCs using a straightforward method (Scheme 1a). The effects of the CNC concentration on the resulting hydrogel properties were systematically investigated. The CNCs, PVA, and borax imparted hydrogel with increased strength and self-healing ability, together with thermal responsiveness. Additionally, the homogeneous distribution of CNCs improved the compatibility between starch/PVA and therefore increased the hydrogel’s stretchability. Owing to these advantages, the prepared hydrogels could be utilized as strain sensors. Overall, this research increased the applications for starch-based hydrogels in sustainable wearable sensors.

**Scheme 1:**
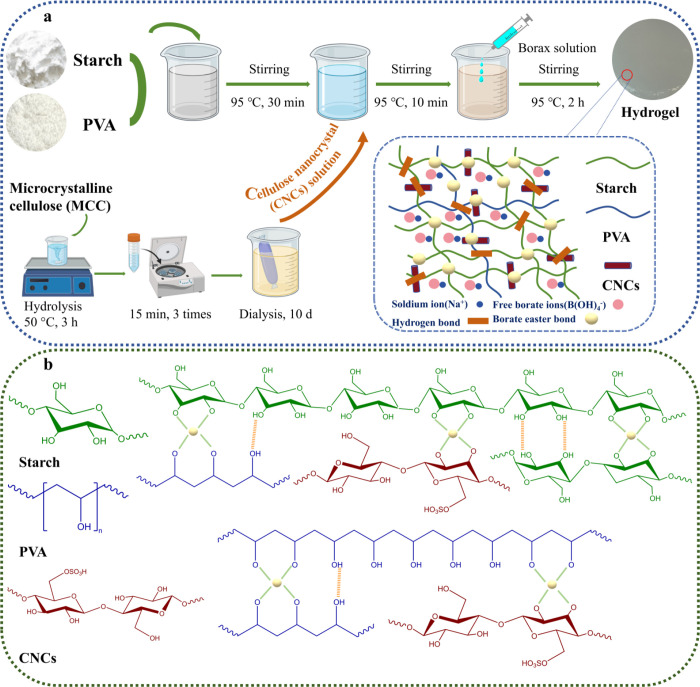
(a) Method of Preparing Starch-Based Dual Reversible Cross-Linking Hydrogels; (b) Chemical Structure and Corresponding Interactions in the Hydrogels

## 2 Experimental Section

### 2.1 Materials

The chemicals used were cornstarch (product code S4126, 27 wt % amylose), poly­(vinyl alcohol) (PVA, product code 341584, *M*_w_ = 89,000–98 000 g/mol, 99+% hydrolyzed), microcrystalline cellulose (20 μm), borax (Na_2_B_4_O_7_·10H_2_O), and sulfuric acid (98%). The double-sided adhesive tape (VHB, 3 M) was purchased from Amazon. All chemicals were purchased from Sigma-Aldrich and used as received.

### 2.2 Preparation of Cellulose Nanocrystal (CNC) Suspension

The suspension were prepared via the acidic hydrolysis method described by Burhani et al.^41^ Microcrystalline cellulose (2.50 g) was added to sulfuric acid (37.5% v/v) (solid/liquid: 1:20), and then, the mixture was continuously magnetically stirred (3 h, 50 °C). Followed by diluting the suspension with distilled water and neutralized via centrifugation (5 times, 4500 rpm, 15 min), the precipitate was subsequently dialyzed via dialysis membranes for 10 days. Finally, the CNC suspension (0.5 g/L) was refrigerated at 6 °C. The morphology of the CNCs (Figure S1) analyzed via transmission electron microscopy (TEM) revealed rod-like morphology (diameter: 11–18 nm, length: 74–200 nm).

### 2.3 Preparation of Starch-Based Hydrogels with Dual Reversible Cross-Linking

Hydrogels were prepared as follows: predesigned weights of starch and PVA were dissolved in distilled water and stirred for 30 min at 95 °C to obtain the completely dissolved starch/PVA solution. A certain amount of the CNC suspension was subsequently added to the starch/PVA mixture and stirred (10 min at 95 °C). Afterward, a freshly prepared borax solution was added at 95 °C and stirred. Also, starch-based dual reversible cross-linking hydrogels were formed when cooled to room temperature. The hydrogels where the amount of CNC suspension added was 0, 1, 3, 5, or 7 mL were named SPB, SPCB-1, SPCB-3, SPCB-5, and SPCB-7 (Table 1), respectively. The hydrogels with starch and PVA without borax or CNCs (labeled with SP) and with borax but without CNCs (labeled with SBP) were utilized as control samples.

**Table 1:** Composition of Hydrogels

**sample**	**cornstarch (g)**	**PVA (g)**	**borax (g)**	**CNC suspension (mL)**
SP	7.50	2.50		
SPB	7.50	2.50	0.50	
SPCB-1	7.50	2.50	0.50	1.00
SPCB-3	7.50	2.50	0.50	3.00
SPCB-5	7.50	2.50	0.50	5.00
SPCB-7	7.50	2.50	0.50	7.00

### 2.4 Fourier Transform Infrared (FTIR) Spectroscopy

FTIR spectra from samples were obtained using a Vertex 70 Bruker spectrometer ranging from 4000 to 400 cm^–1^.

### 2.5 X-ray Diffractometry (XRD)

XRD patterns were analyzed with a Bruker D8 Advance diffractometer with Cu Kα radiation at a 2θ range of 5–50°.

### 2.6 Scanning Electron Microscopy (SEM)

Samples were freeze-dried and coated with a 15 nm layer of Au via a Cressington Sputter Coater 208HR before imaging and then observed via a Philips XL30 field emission gun scanning electron microscope.

### 2.7 Gel Fraction and Swelling Ratio

The gel fraction, swelling ratio, and statistical analyses were performed via the method described in our previous study.^32^ The gel fraction was determined via eq 1, and the swelling ratio was calculated via eq 2:

$$\mathrm{gel}\;\mathrm{fraction}\left(\%\right)=\frac{W_{d}}{W_{i}}\times 100$$ 1

$$\mathrm{swelling}\;\mathrm{ratio}=\frac{W_{s}-W_{d}}{W_{d}}$$ 2

where *W*_i_ and *W*_d_ represent the initial and final dry weight of the hydrogel, respectively, and *W*_s_ represents the weight of the swollen hydrogel. Each experiment was repeated three times.

### 2.8 Thermogravimetric Analysis (TGA)

Thermal analysis was analyzed with a TGA5500 TA Instruments ranging from 30 to 700 °C at a heating rate of 10 °C/min under an N_2_ atmosphere.

### 2.9 Rheological Performance

Rheological performance was evaluated using an Anton Paar MCR302 rheometer. The strain sweep test was performed at 0.1–100% stain. The frequency sweep test was recorded between 0.1 and 100 rad/s.

### 2.10 Mechanical Tests

The mechanical test of the sample (45 mm × 14 mm × 5 mm) was performed via a strain-controlled testing machine (model: Instron 5565) at 10 mm/min. The average of three samples was taken, including the standard deviation.

### 2.11 Self-Healing Capability and Remoldability

The self-healing capability was determined via mechanical and rheological tests. Rheological tests were performed via the method described in our previous study.^32^ For the mechanical tests, the SPCB-5 hydrogel was remolded at room temperature after breaking, after which tensile tests were performed to determine healing efficiency (HE) via eq 3:

$$\mathrm{HE}\left(\%\right)=\frac{S_{A}}{S_{B}}\times 100$$ 3

where *S*_A_ and *S*_B_ are the fracture stress of hydrogels after and before remolding, respectively.

### 2.12 Thermosensitivity Properties

Thermosensitivity was confirmed by rheological tests via a temperature sweep.

### 2.13 Conductivity Characterization

Conductivity of hydrogels was measured via an electrochemical workstation (Biologic SP300) in the frequency range from 1 MHz to 0.1 Hz. The conductivity was determined via eq 4:

$$\sigma =\frac{d}{R\times S}$$ 4

where *d* represents the thickness, *R* stands for the resistance, and *S* stands for the contact area.

### 2.14 Sensing Performance

The sensing performance was evaluated via an Agilent 34410A multimeter. The SPCB-5 hydrogels were fixed on the corresponding skin of a volunteer (e.g., finger, wrist, knee, elbow, leg, and throat) to monitor the motion in different areas of the body. The hydrogels were fixed on the finger and bent according to the Morse code to realize information communication; dots “·” and dashes “-” represent the changes in electrical signals caused by quick and prolonged finger bending, respectively. For the writing application, the hydrogels were connected with copper wires and then sandwiched in poly­(ethylene terephthalate) (PET) films; the changes in the resistance were recorded during writing on the sensor. Additionally, a battery-powered circuit incorporating the SPCB-5 hydrogels into LED indicators was developed to demonstrate their strain sensitivity and healing capabilities. The relative resistance change was determined via eq 5:

$$\frac{\Delta R}{R_{0}}\left(\%\right)=\frac{\left(R-R_{0}\right)}{R_{0}}\times 100$$ 5

where *R* and *R*_0_ are the resistances of the deformed and initial hydrogels, respectively.

The sensitivity and durability were assessed by encapsulating them in the VHB tape to prevent water evaporation, followed by installation on a universal testing machine (Instron 68SC-1, 10 N load cell) connected to a multimeter. Reciprocating cyclic stretching was conducted at various tensile strains and varying stretching speeds. The gauge factor (GF) was determined via eq 6:

$$\mathrm{GF}=\frac{\left(R-R_{0}\right)/R_{0}}{\varepsilon }$$ 6

where ε stands for the applied strain of the hydrogel.

## 3 Results and Discussion

### 3.1 Hydrogel Morphology

The FTIR spectra of native starch, PVA, CNCs, and starch-based conductive hydrogels with dual reversible interactions are shown in Figure 1a. For starch, the 3310 and 2930 cm^–1^ bands arise from the O–H and C–H stretching vibrations, respectively. The 1625 and 1152 cm^–1^ bands correspond to H–O bending and C–O–C stretching, respectively.^42^ For PVA, the 3249 cm^–1^ band corresponds to O–H stretching, whereas those at 2936 and 2906 cm^–1^ stem from the asymmetric and symmetric stretching of CH_2_, respectively.^43^ For CNC, the 1033 cm^–1^ band arises from the sulfate half-ester groups formed during acid hydrolysis.^44^ Moreover, the 1643 cm^–1^ band arises from the adsorbed water within samples.^45^ FTIR spectra of the starch/PVA/CNC hydrogels presented the characteristic absorption peaks of their components. The 1423 and 1333 cm^–1^ bands correspond to B–O–C bonds, suggesting the formation of borate ester bonds between starch, PVA, and CNCs.^46^ Additionally, the broad peak at 3310 cm^–1^ shifts to 3340 cm^–1^ as the CNC concentration increases, indicating the intermolecular hydrogen bonding among starch/PVA/CNC.

**Figure 1:**
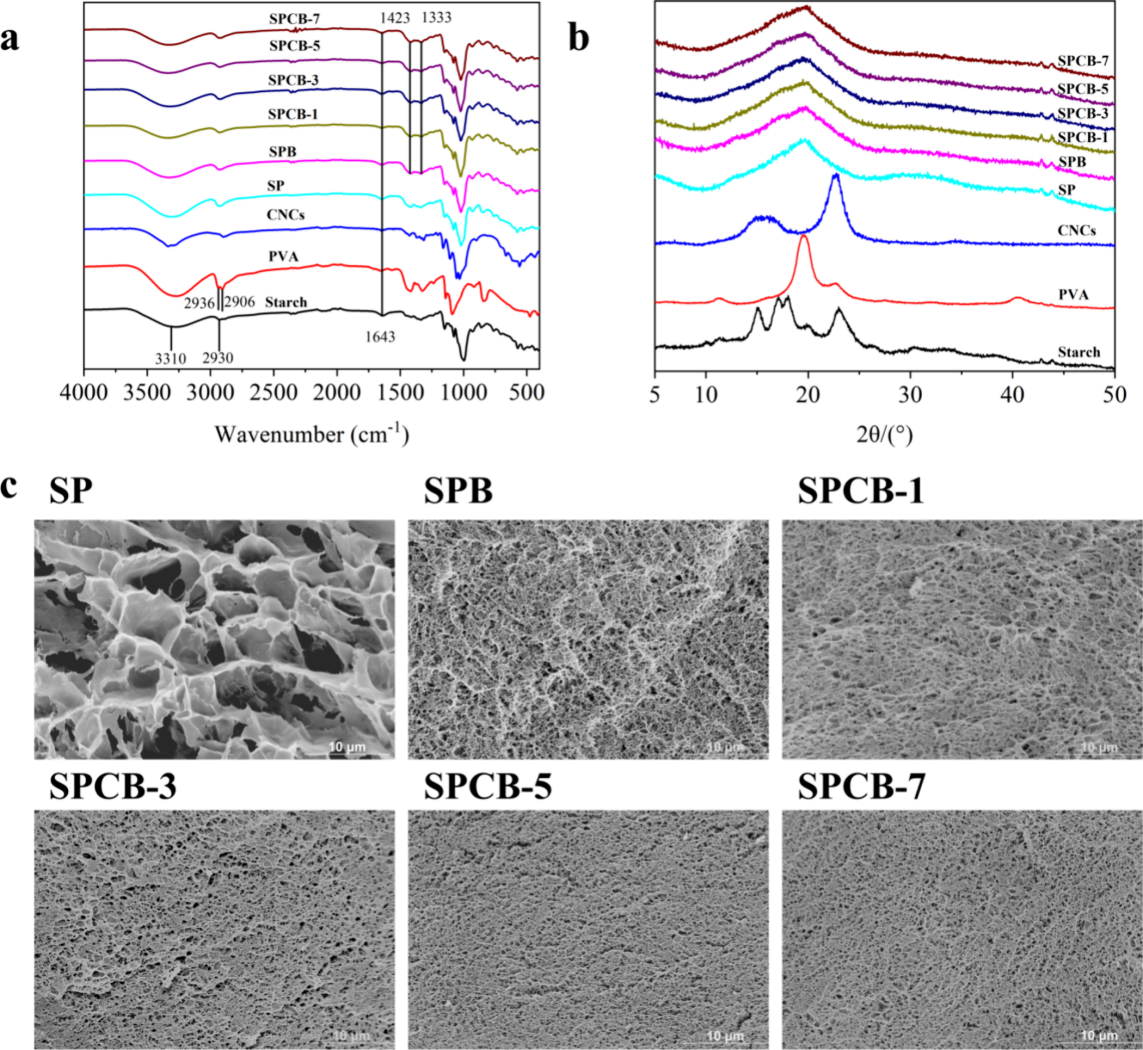
(a) FTIR spectra of sample; (b) XRD patterns of samples; (c) SEM images of freeze-dried hydrogels.

Figure 1b shows the XRD patterns. For native starch, the XRD pattern displays peaks at 15, 17, 18, and 23°, which are typical A-type crystalline structures.^47^ For PVA, peaks at 10.1, 19.6, 22.9, and 40.8° correspond to the (100), (101), (200), and (102) planes of the crystallites, respectively.^48^ For CNCs, peaks at 2θ = 14.5, 16.3, 22.04, and 34.7° correspond to the (101), (101′), (002), and (040) planes of crystalline cellulose, respectively.^49^ After the reaction, hydrogels were formed via hydrogen bonding together with borate ester bonds between the PVA chains, CNCs, and starch chains, as shown in the FTIR results. The consumption of hydroxyl groups within starch could suppress the retrogradation of starch, which led to a decreased crystallinity of the hydrogels. Therefore, the XRD profile showed a broad peak at 20°, which is typical of amorphous starch, and there was no evidence for the diffraction peaks of PVA and CNCs.

Figure 1c shows the morphologies of the hydrogels. During freeze-drying, water in the hydrogel forms ice crystals and then sublimates.^50^ The SP hydrogel had a loose and irregular porous structure due to the weak hydrogel network resulting from the poor compatibility of starch with PVA.^51^ The introduction of borax and CNCs led to a uniformly interconnected network structure, which was formed by densely cross-linked complexes via hydrogen and borate ester bonds. Additionally, CNCs possess many active hydroxyl groups and can form additional bonds and establish hydrogen-bonding interactions within the hydrogel, resulting in a dense network, as confirmed by the FTIR results.^52^ These uniformly distributed pore networks facilitate stable transport channels for Na^+^ and B­(OH)_4_^–^, thereby increasing the electrical conductivity of the hydrogel.

### 3.2 Gel Fraction, Swelling Ratio, and Thermal Stability

As shown in Figure 2a, the gel fraction decreased as CNC concentration increased and then increased since SPCB-5. The numerous active hydroxyl groups of CNCs formed dynamic borate ester bonds with borate ions and hydrogen bonds with starch/PVA chains, thereby weakening the interaction between PVA and starch chains and resulting in a higher soluble fraction of hydrogels. Increasing the CNC content further increased the cross-link density, resulting in a robust hydrogel network.^53^ Figure 2b shows the impact of the CNC concentration on the swelling ratio. The swelling ratio increased as the CNC concentration increased and decreased for SPCB-5 and SPCB-7. The initial increase could be explained by the numerous hydroxyl groups in the CNCs enhancing the hydrophilicity of the hydrogels.^52^ The later decrease in swelling ratio was attributed to the higher cross-link density, which limited the expansion of the network within the hydrogel.

**Figure 2:**
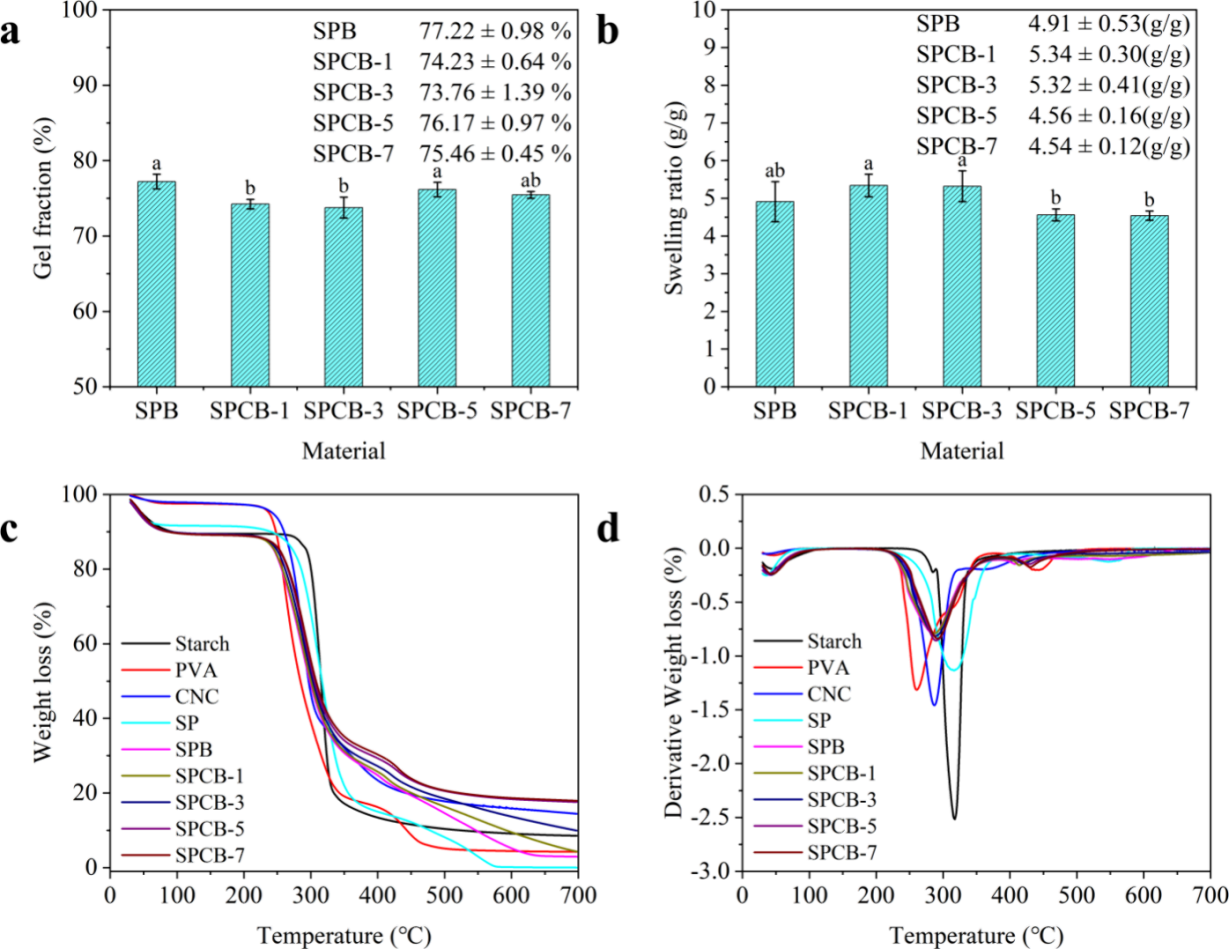
(a) Gel fractions of the hydrogels; (b) swelling ratios of the hydrogels; (c) TGA curves of samples; (d) DTG curves of samples. Values with different letters are significantly different (*p* < 0.05).

Figure 2c,d shows the thermal stabilities for starch, PVA, CNCs, and hydrogels. The native starch exhibited two decomposition stages: water molecule elimination at 25–250 °C and thermal decomposition of the starch backbone at 250–350 °C.^54^ For CNCs, three degradation stages were identified: water evaporation at 25–200 °C, cellulose degradation (including depolymerization, dehydration, and glycosidic unit decomposition) at 200–325 °C, and oxidation and breakdown of charred residues into low-molecular-weight gaseous products at 325–500 °C.^55^ The TGA curves of PVA showed three decomposition stages: water molecule evaporation at 25–200 °C, breakdown of PVA macromolecular chains at 200–380 °C, and disintegration of PVA main chains at 380–500 °C.^56^ The TGA curves of the hydrogels revealed three decomposition stages at 25–250, 250–380, and 380–500 °C. The second-stage peak decomposition temperature of hydrogels decreased, which was due to the disruption of the crystalline structure during gelatinization.^57^ In addition, hydrogels with CNCs presented a higher peak decomposition temperature than those with PVA or CNCs did, which was lower than that of the SP hydrogel, indicating good compatibility among starch, PVA, and CNCs. Moreover, the increased weight residue of the hydrogels after heating indicates that CNCs enhance their thermal stability.

### 3.3 Rheological Properties

Figure 3a,b shows the *G*′ and *G*″ curves relative to the strain amplitude (γ = 0.1–100%) at ω = 10 rad/s. To ensure that each test was performed in a linear viscoelastic region, γ = 1% was applied since the *G*′ values were relatively stable.^58^ The consistently higher *G*′ than *G*″ indicated the elastic-like behavior of all of the hydrogels. Among all of the hydrogels, SPCB-7 exhibited the highest Gmax′ value of approximately 3809.4 Pa, which was 8.7-fold greater than that of the SP hydrogel (∼440.07 Pa), indicating the significant reinforcing effect of borax and CNCs. In addition, *G*′ and *G*″ increased with increasing CNC concentration, from 3291.5 Pa (*G*′) and 1100.4 Pa (*G*″) for the SPB hydrogel to 3809.4 Pa (*G*′) and 1259.9 Pa (*G*″) for the SPCB-7 hydrogel. This could be explained by the numerous hydroxyl groups of CNCs that formed many hydrogen bonds with PVA and starch and borate ester bonds with borax, leading to a stiffer gel network structure with a greater mechanical strength. For SPCB-3 and SPCB-5 hydrogels, their *G*′ and *G*″ were lower than the SPCB-1 hydrogel and higher than the SPB hydrogel. There exists a competition between the borate ester bonds and hydrogen bonds, since the hydroxyl groups of starch/PVA formed hydrogen bonds with CNCs and therefore decreased the number of hydroxyl groups that formed borate ester bonds with borax, resulting in a weaker hydrogel.

**Figure 3:**
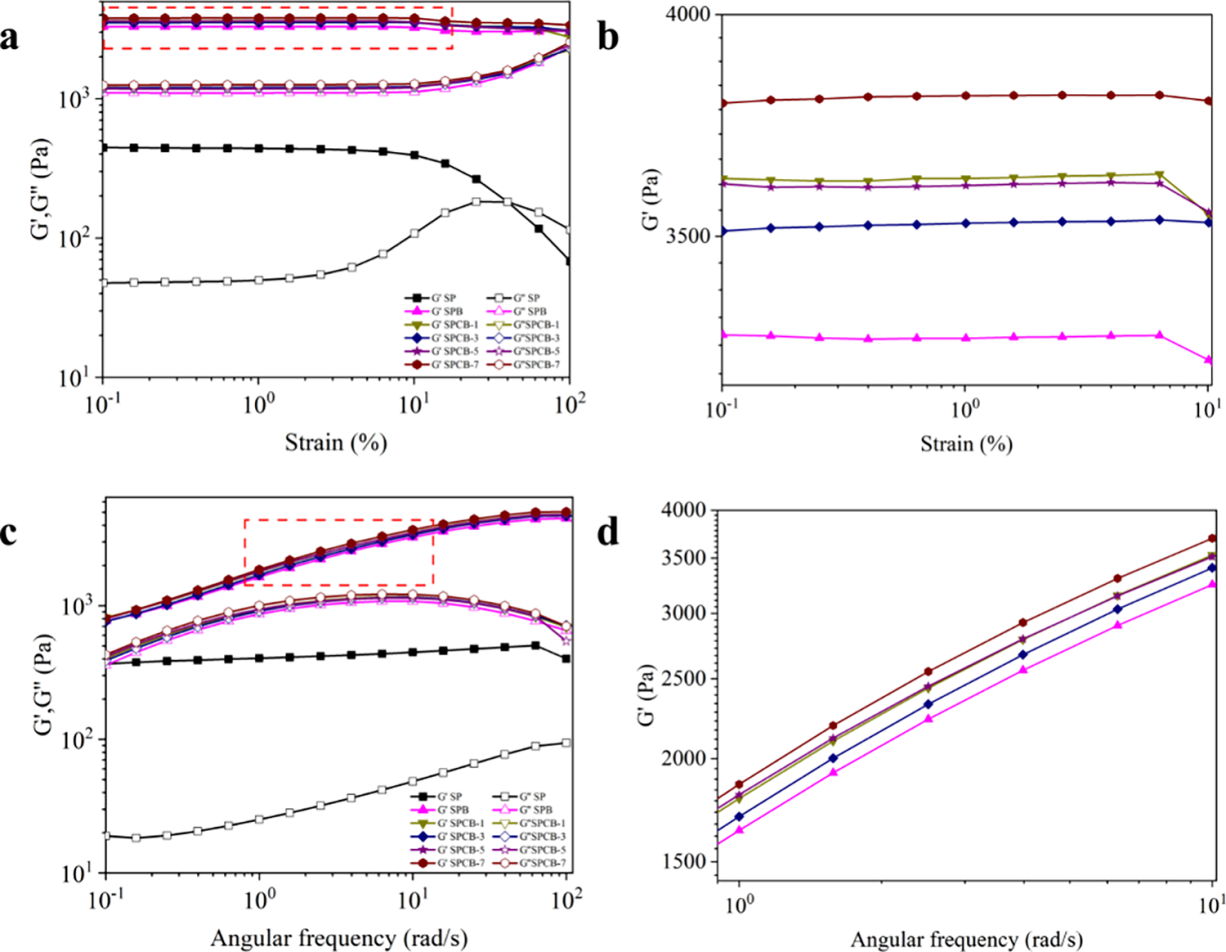
(a) Strain sweep curves; (b) enlargement of the region marked in red in panel (a); (c) frequency sweep curves; (d) enlargement of the region marked in red in panel (c).

Figure 3c,d illustrates frequency sweep curves of hydrogels. Compared with permanently cross-linked hydrogels, all hydrogels exhibited frequency-dependent moduli, indicating dynamic cross-linking.^59^ In addition, the G′ values increased until reaching a plateau, indicating that the polymer was entangled.^39^ Furthermore, the G′ and G″ trends mirrored those of the strain sweep tests. These findings verified the existence of chain entanglements and dual reversible interactions within the hydrogels.

### 3.4 Mechanical Performance

Figure 4a shows that adding CNCs significantly enhanced the strength of the hydrogels, which is consistent with the rheological properties. The tensile fracture stress, strain, and toughness of the SPCB-5 hydrogel were 0.538 ± 0.016 kPa, 304.71 ± 4.79%, and 5.99 ± 0.72 kJ/m^3^, respectively, which were significantly greater than those of the SPB hydrogel (0.270 ± 0.014 kPa, 210.64 ± 12.36%, and 1.93 ± 0.15 kJ/m^3^, respectively). This may be due to the hydrophilic groups on the CNC surface forming hydrogen bonds with PVA and starch chains and borate ester bonds with borax that act as noncovalent or dynamic covalent sacrificial bonds to dissipate energy efficiently.^24,60^ As shown in Figure 4b, the SPCB-5 hydrogel could be stretched from 2.5 to 26 cm without breaking, demonstrating greater toughness and superior tensile properties than the SPB hydrogel, which broke when stretched. This behavior is related to the dual reversible interactions within the hydrogels that effectively dissipate external energy.

**Figure 4:**
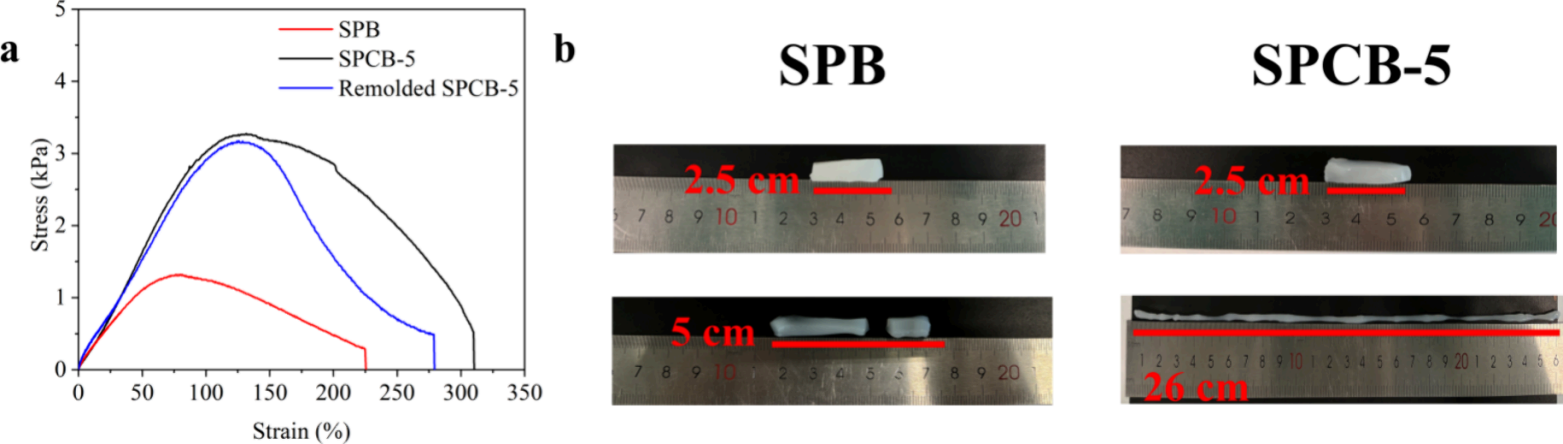
(a) Tensile stress–strain curves of hydrogels. (b) Pictures of the manually stretched SPB and SPCB-5 hydrogels.

### 3.5 Self-Healing and Reprocessability

The self-healing ability is a highly desirable feature of a hydrogel, as it allows the material to recover its mechanical integrity after sustaining damage. In this study, SPCB-5 was chosen as the representative hydrogel. As depicted in Figure 5a, the SPCB-5 hydrogel was cut into four sections and contacted at room temperature. Remarkably, the four segments autonomously adhered to each other and self-healed without any external force or stimulus. Figure 5b shows the changes in *G*′ and *G*″ for the original and self-healed SPCB-5 hydrogels, demonstrating their effective restoration of the mechanical properties. The *G*′ and *G*″ of the self-healed and original hydrogel were similar, indicating the restoration of its internal structure. Figure 5c shows the self-healing recovery under strain damage. The SPCB-5 hydrogel was solid at a low strain, with a *G*′ of 2921.8 Pa and a *G*″ of 987.78 Pa. The *G*′ subsequently decreased to 703.24 Pa, which was lower than *G*″ (1280.4 Pa) under high strain (γ = 150%), suggesting the transient transition of the hydrogel from a quasisolid state to a quasiliquid state.^39^ Upon reducing γ, *G*′ and *G*″ immediately recovered, suggesting its inherent self-recovery capability. As depicted in Figure 4a, the SPCB-5 hydrogel also displayed remoldability. After the tensile test, the sample was remolded into a rectangle. The fracture toughness decreased from 5.99 ± 0.72 to 4.92 ± 0.72 kJ/m^3^. The remolded hydrogel exhibited a fracture stress of 0.489 kPa, with a healing efficiency reaching 91.03%. These findings demonstrated the hydrogel’s excellent self-healing capacity and remoldability owing to dual reversible cross-linking of hydrogen bonds and borate ester bonds among the hydroxyl groups of the PVA chains, CNCs, and starch chains.

**Figure 5:**
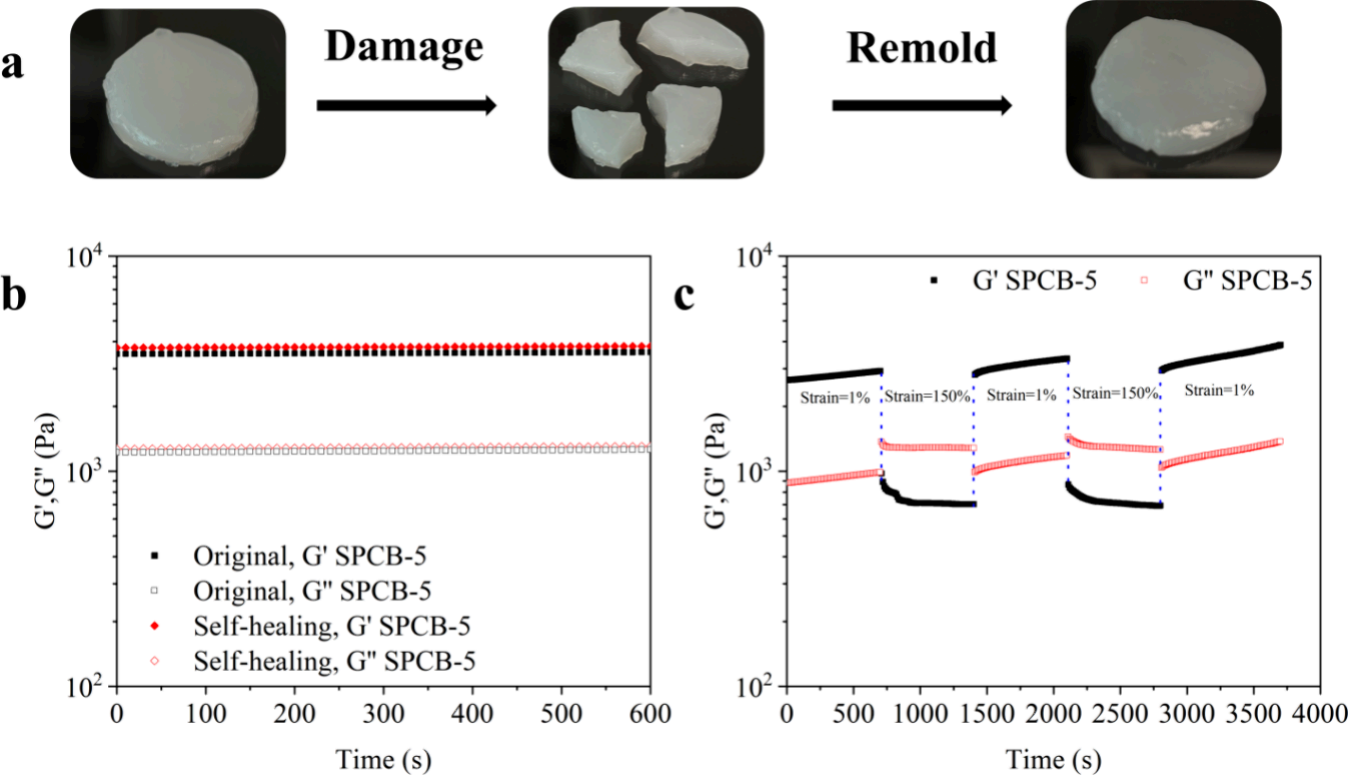
(a) Photographs of the SPCB-5 hydrogel demonstrating self-healing; (b) *G*′ and *G*″ as for the original and self-healed SPCB-5 hydrogels; (c) continuous step-strain measurements of the SPCB-5 hydrogel.

### 3.6 Thermosensitivity Properties

Figure 6 shows the thermosensitivity of the SPCB-5 hydrogel. During the first heating, *G*′ decreased dramatically from 1338.1 to 126.32 Pa with increasing temperature from 20 to 95 °C. After cooling, *G*′ of the hydrogel increased, demonstrating its recovery ability. During the second heating, *G*′ decreased again when the temperature increased. These findings reflect the hydrogel’s thermosensitivity, attributed to the reversible and exothermic interactions between borate ions and the hydroxyl groups of CNCs, PVA, and starch.^23^

**Figure 6:**
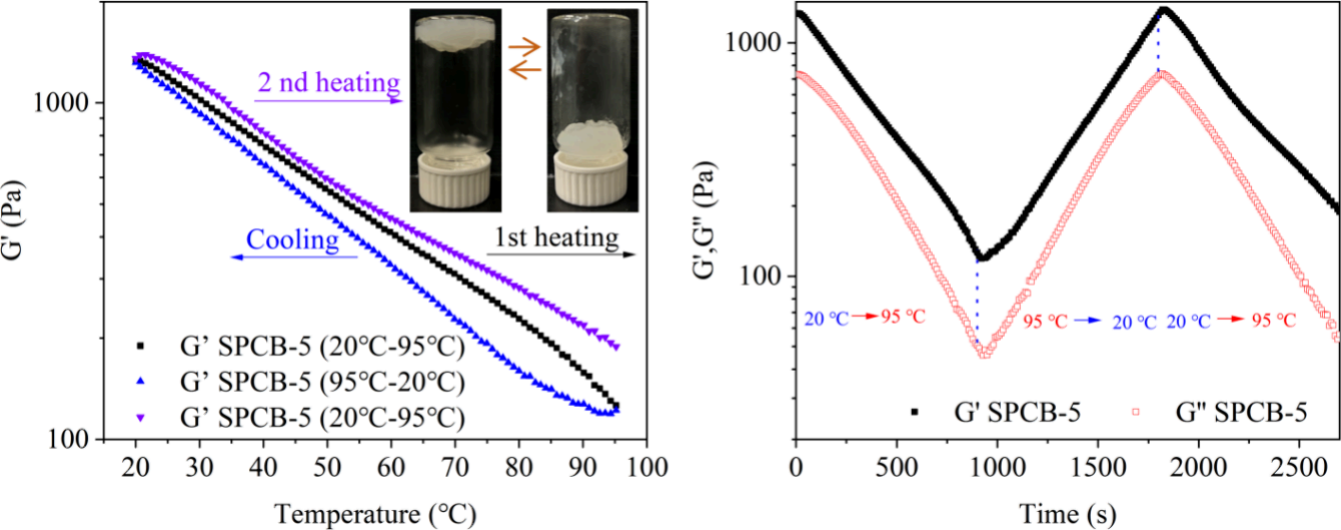
Thermosensitivity of the SPCB-5 hydrogel.

### 3.7 Electrical Property and Sensor Application

The hydrogels showed evident ionic conductivity because of the Na^+^ and B­(OH)_4_^–^ within the network. Figure 7a,b shows the EIS spectra and ionic conductivity, and the ionic conductivity of the SPCB-5 hydrogel was 23.3 mS/cm, which was significantly greater than that of the SPB hydrogel (14.7 mS/cm). The enhanced ionic conductivity may be due to the homogeneous distribution of CNCs in SPCB-5, which enhances the compatibility between starch, PVA, and CNCs that facilitates stable transport channels for ion transportation within the hydrogels. In addition, the conductivity of the SPCB-5 hydrogels after healing and hydrogels after 5 and 20 stretching cycles showed only a slight decrease. These results demonstrate its reliability and self-healing capacity.

**Figure 7:**
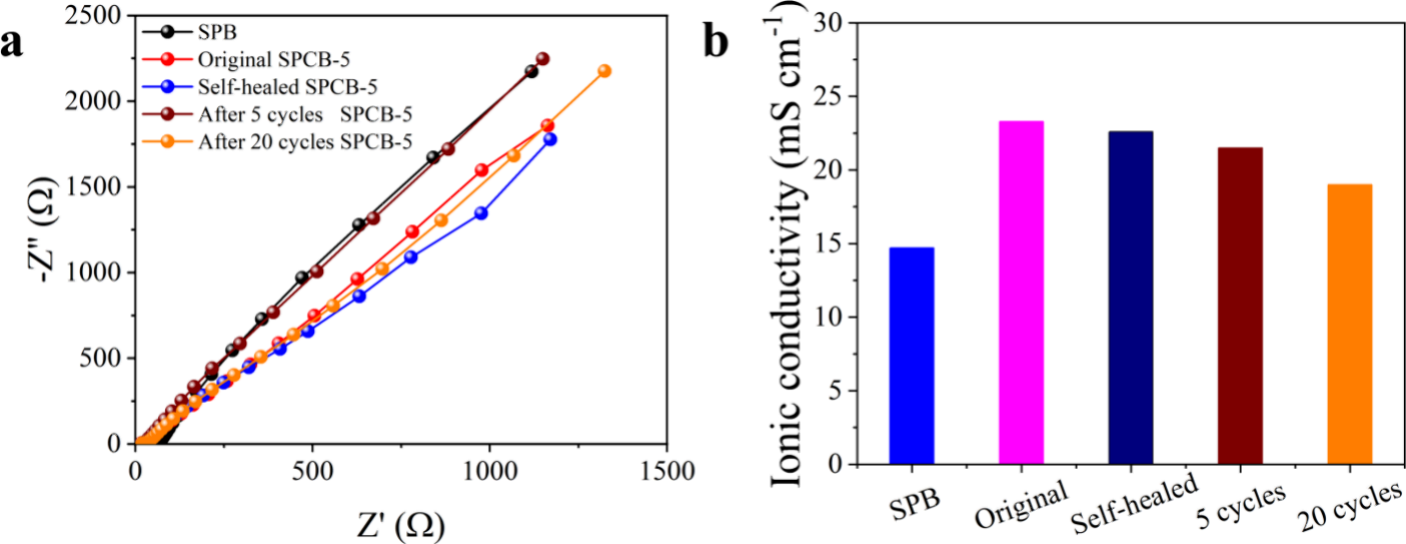
(a) EIS spectra of SPB, original, and self-healed SPCB-5 hydrogels and the SPCB-5 hydrogel after different stretching cycles. (b) Corresponding ionic conductivity of SPB, original, and self-healed SPCB-5 hydrogels and the SPCB-5 hydrogel after different stretching cycles.

The excellent stretchability as well as self-healing properties of the SPCB-5 hydrogel make it suitable as a sensor that can monitor various human motions. Figure 8 shows the application of an SPCB-5 hydrogel sensor in monitoring both significant and subtle human movements, with Figure 8a–e showing the relative resistance change (Δ*R*/*R*_0_) during the bending and unbending of various joints including the arm, wrist, fingers, neck, and leg. The hydrogel sensors can produce consistent and stable electrical signals, demonstrating their reliability. Compared with the SPB hydrogel, the SPCB-5 hydrogel exhibited significantly greater strain sensitivity (Figure S2), with greater Δ*R*/*R*_0_ values observed during arm bending (approximately 30 vs 6%), wrist bending (20 vs 5%), and finger bending (40 vs 10%). This highlights the superior strain sensitivity of the SPCB-5 hydrogel. The homogeneous distribution of CNCs in SPCB-5 enhances the compatibility between starch, PVA, and CNCs through dual reversible interactions, which improves the stretchability of the hydrogel.

**Figure 8:**
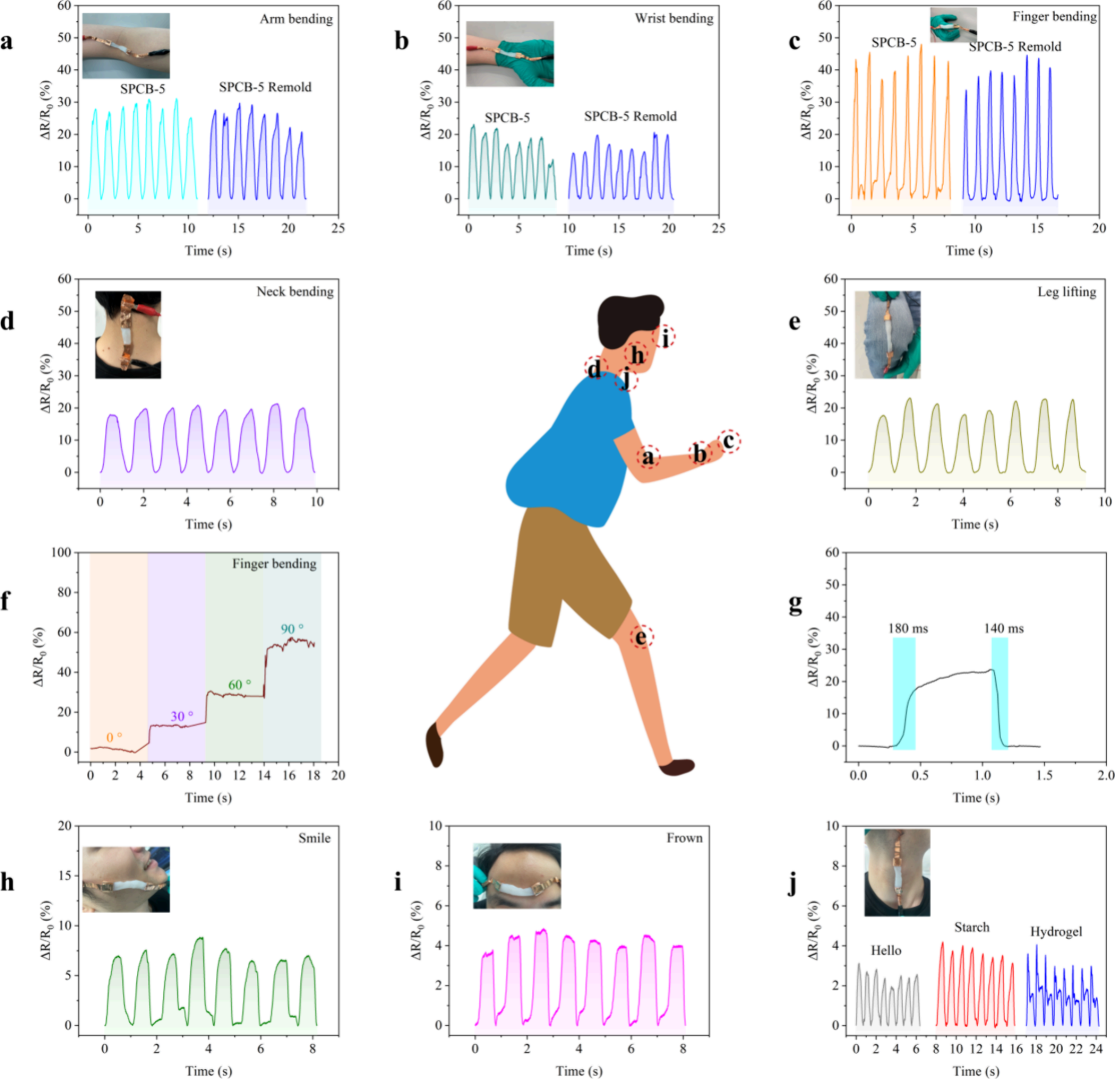
Application demonstration of strain sensors based on the SPCB-5 hydrogel: (a) arm bending; (b) wrist bending; (c) finger bending; (d) neck bending; (e) leg lifting; (f) bending of the finger at 0, 30, 60, and 90°; (g) quick response of the hydrogel sensor to bending; (h) smile; (i) crown; (j) pronouncing (hello, starch, and hydrogel). (Inset: strain sensor attached to the corresponding body part.) All the experiments were performed at the University of Groningen (5613.0091).

Furthermore, the hydrogel demonstrates excellent reusability, as evidenced by Figure 8a–c, which shows that the Δ*R*/*R*_0_ values for the arm, wrist, and finger bending of the remolded SPCB-5 hydrogel closely match the Δ*R*/*R*_0_ values of the original hydrogel. Figure 8f exhibits the stepwise change in relative resistance (0–60%) as the bending angle increases from 0 to 90°. Figure 8g shows that the SPCB-5 hydrogel has a response time of 180 ms for bending and a recovery time of 140 ms, demonstrating its suitability for rapid monitoring of transient human motions.^61^ Figure 8h–j illustrates that the Δ*R*/*R*_0_ of the SPCB-5 hydrogel induced by small movements, such as frowning and smiling, was less than 10%.^62^ In addition, the sensor produced a distinct and consistent electrical signal when placed near the throat as the volunteer spoke different words, such as “hello”, “starch”, and “hydrogel”.

Figure 9b shows the durability of the SPCB-5 hydrogel; the hydrogel exhibited good stability over 5000 cycles at 30% strain. As shown in Figure 9c, the GF was divided into three regions. The GF was 0.87 for the strain (0–40%), increased to 0.95 for the strain (40–80%), and then decreased to 0.50 with increasing strain (80–110%), revealing its high sensitivity. Figure 9d shows a rapid increase in the Δ*R*/*R*_0_ as the strain changed from 10 to 80%, indicating a wide operating range of the sensor. Figure 9e shows that Δ*R*/*R*_0_ had the same amplitude when the frequency increased from 0.1 to 0.5 Hz, which confirmed the wide adaptability of the sensor at different frequencies. These findings demonstrated the reliability and high sensitivity of the sensors, which are important for practical applications.

**Figure 9:**
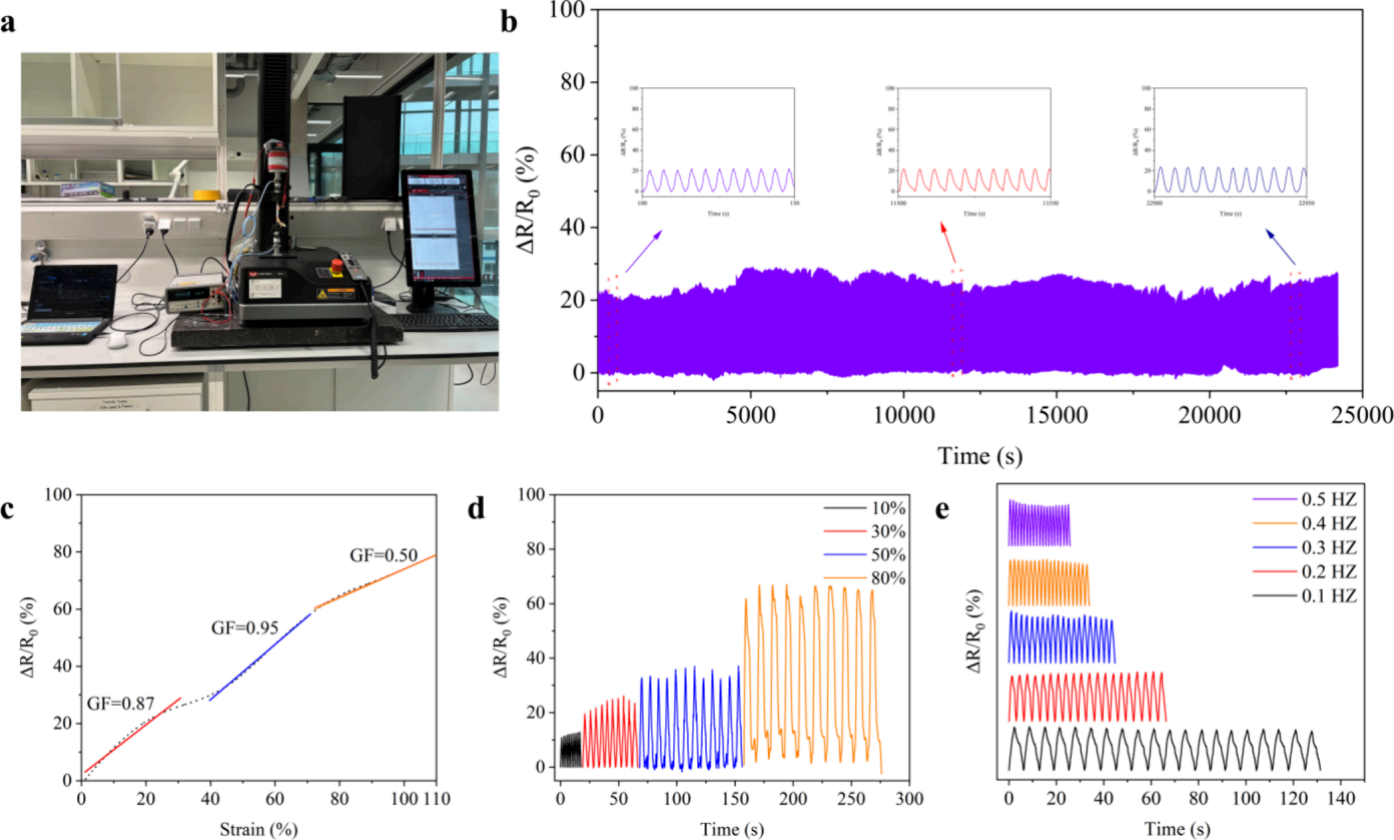
(a) Photograph of the sensing tests; (b) durability test during 5000 cycles at 30% tensile strain (inset: amplified signal at 100–150, 11,500–11,550, and 22,900–22,950 s); (c) Δ*R*/*R*_0_ versus tensile strain; (d) Δ*R*/*R*_0_ under various tensile strains (10, 30, 50, and 80%); (e) Δ*R*/*R*_0_ at different frequencies at 30% tensile strain.

Figure 10a shows that the conductivity of the SPCB-5 hydrogel can be monitored with an LED, which lights up when the hydrogel is integrated into the circuit, indicating good conductivity. As hydrogels were stretched from 0 to 100%, the brightness significantly decreased because of the increased resistance. Additionally, when they were remolded, the brightness was similar to that before repair. These results further confirmed that the SPCB-5 hydrogel had excellent self-healing properties as well as electrical conductivity. Additionally, the SPCB-5 hydrogel was explored for use in information communication via the Morse code. The Morse code is a recognized encryption and decryption tool that uses dashes and dots to represent English letters.^63^ Figure 10b shows a diagram of the Morse code. Figure 10c–j shows the Δ*R*/*R*_0_ of the SPCB-5 hydrogel sending information such as “SOS”, “RUG”, “HELLO”, “HELP”, and “LOVE”. The repeatable electrical signal outputs demonstrated the potential of the SPCB-5 hydrogel as a Morse code generator to aid communication for people with speech or writing difficulties. Figure 10h shows a diagram of the writing sensor, which was fabricated by connecting the SPCB-5 hydrogel with copper wires and then sandwiching it with poly­(ethylene terephthalate) (PET) films. As shown in Figure 10i–m, the sensor can respond to the words “Hi”, “Hello”, and different letters “R”, “U”, and “G”, with a clear and stable electrical signal waveform for the same letter in four repetitive cycles, revealing the potential use of the SPCB-5 hydrogel for smart handwriting recognition.

**Figure 10:**
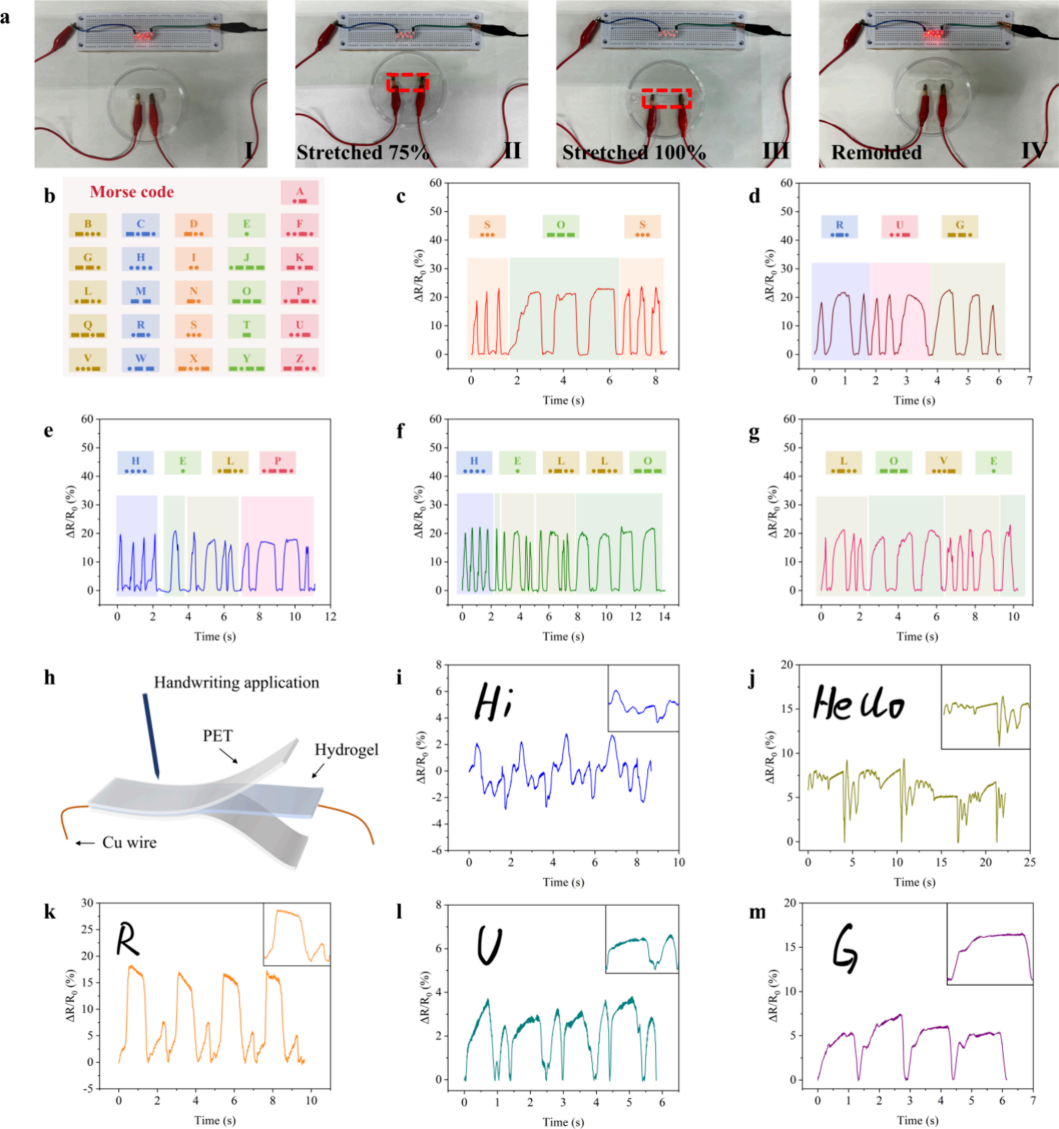
(a) LED lightness: (I) initial, (II) stretched (75%), (II) stretched (100%), and (IV) remolded; (b) diagram of the Morse code; (c) Morse code transmission “SOS”; (d) Morse code transmission “RUG”; (e) Morse code transmission “HELLO”; (f) Morse code transmission “HELP”; (g) Morse code transmission “LOVE”; (h) diagram of the writing sensor; relative resistance changes of writing (i) “Hi”, (j) “Hello”, (k) “R”, (l) “U”, and (m) “G”.

## 4 Conclusions

In general, we synthesized conductive hydrogels using biocompatible raw materials involving starch, CNCs, and PVA by utilizing borax as a cross-linker through a straightforward one-pot method. The hydrogels demonstrated improved mechanical strength, thermal responsiveness, and self-healing capabilities via the synergistic dual reversible cross-linking of hydrogen bonds and borate ester bonds among the PVA chains, CNCs, and starch chains. The introduction of CNCs increased the tensile strength, stretchability, and strain sensitivity of the prepared hydrogel; the tensile strain of the hydrogel with CNCs was almost doubled in comparison to that of the hydrogel without CNCs. In addition, our hydrogels exhibit conductivity and sensitivity due to the Na^+^ and B­(OH)_4_^–^ within the uniform internal network structure. The prepared hydrogels can be utilized as wearable strain sensors to precisely monitor different human movements, including small actions (expression changes and pronunciation) and large movements (the bending movement of joints), to sense handwriting and detect Morse code. Moreover, the hydrogels could be remolded at room temperature, demonstrating their flexibility and circularity, which are essential for practical applications. On the basis of these outstanding properties, this study broadens the application of starch-based hydrogels in sustainable wearable sensors.

## Supplementary Material



## Data Availability

The raw data are available upon request.

## Notes

Additional experimental results: TEM micrograph of CNC and application demonstration of strain sensors based on the SPB hydrogel (PDF)

## References

[1] He T., Wang H., Wang J., Tian X., Wen F., Shi Q., Ho J. S., Lee C. (2019). Self-Sustainable Wearable Textile
Nano-Energy Nano-System (NENS) for Next-Generation Healthcare Applications. Adv. Sci..

[2] Zhao Y. S., Zhang B. Z., Yao B. W., Qiu Y., Peng Z. H., Zhang Y. C., Alsaid Y., Frenkel I., Youssef K., Pei Q. B. (2020). Hierarchically Structured
Stretchable Conductive Hydrogels
for High-Performance Wearable Strain Sensors and Supercapacitors. Matter.

[3] Liu S. J., Li L. (2017). Ultrastretchable and Self-Healing
Double-Network Hydrogel for 3D
Printing and Strain Sensor. ACS Appl. Mater.
Interfaces.

[4] Zheng H. Y., Lin N., He Y. Y., Zuo B. Q. (2021). Self-Healing, Self-Adhesive Silk
Fibroin Conductive Hydrogel as a Flexible Strain Sensor. ACS Appl. Mater. Interfaces.

[5] Chen Q., Li W. J., Yan F., Maniar D., van Dijken J., Rudolf P., Pei Y. T., Loos K. (2023). Lightweight Triboelectric
Nanogenerators Based on Hollow Stellate Cellulose Films Derived from
Juncus effusus L. Aerenchyma. Adv. Funct. Mater..

[6] Do N. H. N., Truong Q. T., Le P. K., Ha A. C. (2022). Recent
developments
in chitosan hydrogels carrying natural bioactive compounds. Carbohydr. Polym..

[7] Zeng S., Zhang J. Y., Zu G. Q., Huang J. (2021). Transparent, flexible,
and multifunctional starch-based double-network hydrogels as high-performance
wearable electronics. Carbohydr. Polym..

[8] Pan X. S., Li J., Ma N., Ma X. J., Gao M. (2023). Bacterial cellulose
hydrogel for sensors. Chem. Eng. J..

[9] Lv Z., Liu J. Z., Yang X., Fan D. Y., Cao J., Luo Y. Y., Zhang X. X. (2020). Naturally Derived Wearable Strain
Sensors with Enhanced Mechanical Properties and High Sensitivity. ACS Appl. Mater. Interfaces.

[10] Elkaliny N. E., Alzamel N. M., Moussa S. H., Elodamy N. I., Madkor E. A., Ibrahim E. M., Elshobary M. E., Ismail G. A. (2024). Macroalgae Bioplastics:
A Sustainable Shift to Mitigate the Ecological Impact of Petroleum-Based
Plastics. Polymers.

[11] Chen Q., van Dijken J., Maniar D., Loos K. (2023). Aerenchyma tissue of
Juncus effusus L.: a novel resource for sustainable natural cellulose
foams. Cellulose.

[12] Burhani D., Voet V. S. D., Folkersma R., Maniar D., Loos K. (2024). Potential
of Nanocellulose for Microplastic removal: Perspective and challenges. Tetrahedron Green Chem..

[13] Boetje L., Lan X. H., Silvianti F., van Dijken J., Polhuis M., Loos K. (2022). A more efficient synthesis
and properties
of saturated and unsaturated starch esters. Carbohydr. Polym..

[14] Lan X. H., Li W. J., Ye C. N., Boetje L., Pelras T., Silvianti F., Chen Q., Pei Y. T., Loos K. (2023). Scalable and
Degradable Dextrin-Based Elastomers for Wearable Touch Sensing. ACS Appl. Mater. Interfaces.

[15] Lu K., Zhu J., Bao X. Y., Liu H. S., Yu L., Chen L. (2020). Effect of
starch microstructure on microwave-assisted esterification. Int. J. Biol. Macromol..

[16] Hu J. T., Ma N., Fu X., Zhang S. B., Liu H. S., Liu F. (2022). Developing
DHA microcapsules using linear dextrin aggregates of different chain
length distributions. Carbohydr. Polym..

[17] Ahmadi-Abhari S., Woortman A. J. J., Hamer R. J., Loos K. (2013). Assessment of the influence
of amylose-LPC complexation on the extent of wheat starch digestibility
by size-exclusion chromatography. Food Chem..

[18] Ciric J., Loos K. (2013). Synthesis of branched polysaccharides with tunable degree of branching. Carbohydr. Polym..

[19] Yassaroh Y., Woortman A. J. J., Loos K. (2021). Physicochemical
properties of heat-moisture
treated, stearic acid complexed starch: The effect of complexation
time and temperature. Int. J. Biol. Macromol..

[20] Yassaroh Y., Nurhaini F. F., Woortman A. J. J., Loos K. (2021). Physicochemical properties
of heat-moisture treated, sodium stearate complexed starch: The effect
of sodium stearate concentration. Carbohydr.
Polym..

[21] Lu B. L., Lin F. C., Jiang X., Cheng J. J., Lu Q. L., Song J. B., Chen C., Huang B. (2017). One-Pot Assembly of
Microfibrillated Cellulose Reinforced PVA-Borax Hydrogels with Self-Healing
and pH-Responsive Properties. ACS Sustain Chem.
Eng..

[22] Bagri L. P., Bajpai J., Bajpai A. K. (2009). Cryogenic Designing
of Biocompatible
Blends of Polyvinyl alcohol and Starch with Macroporous Architecture. J. Macromol. Sci. Part A-Pure Appl. Chem..

[23] Huang S. Q., Su S. Y., Gan H. B., Wu L. J., Lin C. H., Xu D. Y., Zhou H. F., Lin X. L., Qin Y. L. (2019). Facile
fabrication and characterization of highly stretchable lignin-based
hydroxyethyl cellulose self-healing hydrogel. Carbohydr. Polym..

[24] Han J. Q., Lei T. Z., Wu Q. L. (2013). Facile
preparation of mouldable polyvinyl
alcohol-borax hydrogels reinforced by well-dispersed cellulose nanoparticles:
physical, viscoelastic and mechanical properties. Cellulose.

[25] Cosse R. L., van den Berg T., Voet V., Folkersma R., Loos K. (2024). Innovative Approaches for Manufacturing Epoxy-Modified Wood and Cellulose
Fiber Composites: A Comparison between Injection Molding and 3D Printing. ChemPlusChem.

[26] Moon R. J., Martini A., Nairn J., Simonsen J., Youngblood J. (2011). Cellulose
nanomaterials review: structure, properties and nanocomposites. Chem. Soc. Rev..

[27] Mariano M., El Kissi N., Dufresne A. (2014). Cellulose
Nanocrystals and Related
Nanocomposites: Review of some Properties and Challenges. J. Polym. Sci. Pt. B-Polym. Phys..

[28] Liao H., Su J., Han J., Xiao T., Sun X., Cui G., Duan X., Shi P. (2024). An Intrinsic Self-Healable, Anti-Freezable
and Ionically Conductive Hydrogel for Soft Ionotronics Induced by
Imidazolyl Cross-Linker Molecules Anchored with Dynamic Disulfide
Bonds. Macromol. Rapid Commun..

[29] Shao C. Y., Wang M., Chang H. L., Xu F., Yang J. (2017). A Self-Healing
Cellulose Nanocrystal-Poly­(ethylene glycol) Nanocomposite Hydrogel
via Diels-Alder Click Reaction. ACS Sustain
Chem. Eng..

[30] Chao A., Negulescu J., Zhang D. H. (2016). Dynamic Covalent
Polymer Networks
Based on Degenerative Imine Bond Exchange: Tuning the Malleability
and Self-Healing Properties by Solvent. Macromolecules.

[31] Pepels M., Filot I., Klumperman B., Goossens H. (2013). Self-healing systems
based on disulfide-thiol exchange reactions. Polym. Chem..

[32] Lu K., Folkersma R., Voet V. S. D., Loos K. (2024). Effects of the Amylose/Amylopectin
Ratio of Starch on Borax-Crosslinked Hydrogels. Polymers.

[33] Ghoorchian A., Simon J. R., Bharti B., Han W., Zhao X. H., Chilkoti A., López G. P. (2015). Bioinspired
Reversibly Cross-linked
Hydrogels Comprising Polypeptide Micelles Exhibit Enhanced Mechanical
Properties. Adv. Funct. Mater..

[34] Nakahata M., Takashima Y., Yamaguchi H., Harada A. (2011). Redox-responsive self-healing
materials formed from host-guest polymers. Nat.
Commun..

[35] Luo F., Sun T. L., Nakajima T., Kurokawa T., Zhao Y., Sato K., Ihsan A. B., Li X., Guo H., Gong J. P. (2015). Oppositely Charged Polyelectrolytes Form Tough, Self-Healing,
and Rebuildable Hydrogels. Adv. Mater..

[36] Chen Y. L., Guan Z. B. (2014). Multivalent hydrogen
bonding block copolymers self-assemble
into strong and tough self-healing materials. Chem. Commun..

[37] Seidi F., Jin Y. C., Han J. Q., Saeb M. R., Akbari A., Hosseini S. H., Shabanian M., Xiao H. N. (2020). Self-healing Polyol/Borax
Hydrogels: Fabrications, Properties and Applications. Chem. Rec..

[38] Mate C. J., Mishra S. (2020). Synthesis of borax
cross-linked Jhingan gum hydrogel
for remediation of Remazol Brilliant Blue R (RBBR) dye from water:
Adsorption isotherm, kinetic, thermodynamic and biodegradation studies. Int. J. Biol. Macromol..

[39] Ding Q. Q., Xu X. W., Yue Y. Y., Mei C. T., Huang C. B., Jiang S. H., Wu Q. L., Han J. Q. (2018). Nanocellulose-Mediated
Electroconductive Self-Healing Hydrogels with High Strength, Plasticity,
Viscoelasticity, Stretchability, and Biocompatibility toward Multifunctional
Applications. ACS Appl. Mater. Interfaces.

[40] Wang Y. L., Huang H. L., Wu J. L., Han L., Yang Z. L., Jiang Z. C., Wang R., Huang Z. J., Xu M. (2020). Ultrafast
Self-Healing, Reusable, and Conductive Polysaccharide-Based Hydrogels
for Sensitive Ionic Sensors. ACS Sustain Chem.
Eng..

[41] Burhani D., Septevani A. A., Setiawan R., Djannah L. M., Putra M. A., Kusumah S. S., Sondari D. (2021). Self-Assembled Behavior
of Ultralightweight
Aerogel from a Mixture of CNC/CNF from Oil Palm Empty Fruit Bunches. Polymers.

[42] Kumar K., Loos K. (2019). Deciphering Structures
of Inclusion Complexes of Amylose with Natural
Phenolic Amphiphiles. ACS Omega.

[43] Jipa I., Stoica A., Stroescu M., Dobre L. M., Dobre T., Jinga S., Tardei C. (2012). Potassium sorbate release from poly­(vinyl
alcohol)-bacterial cellulose films. Chem. Pap..

[44] Suganuma S., Nakajima K., Kitano M., Yamaguchi D., Kato H., Hayashi S., Hara M. (2008). Hydrolysis
of Cellulose
by Amorphous Carbon Bearing SO3H, COOH, and OH Groups. J. Am. Chem. Soc..

[45] Vu A. N., Nguyen L. H., Tran H. C. V., Yoshimura K., Tran T. D., Van Le H., Nguyen N. U. T. (2024). Cellulose
nanocrystals
extracted from rice husk using the formic/peroxyformic acid process:
isolation and structural characterization. RSC
Adv..

[46] Spoljaric S., Salminen A., Luong N. D., Seppälä J. (2014). Stable, self-healing
hydrogels from nanofibrillated cellulose, poly­(vinyl alcohol) and
borax via reversible crosslinking. Eur. Polym.
J..

[47] Yassaroh Y., Woortman A. J. J., Loos K. (2019). A new way
to improve physicochemical
properties of potato starch. Carbohydr. Polym..

[48] Chen Y. N., Jiao C., Zhao Y. X., Zhang J. A., Wang H. L. (2018). Self-Assembled
Polyvinyl Alcohol Tannic Acid Hydrogels with Diverse Microstructures
and Good Mechanical Properties. ACS Omega.

[49] Olad A., Doustdar F., Gharekhani H. (2020). Fabrication and characterization
of a starch-based superabsorbent hydrogel composite reinforced with
cellulose nanocrystals from potato peel waste. Colloids Surf., A.

[50] Wu K., Fang Y., Wu H. X., Wan Y., Qian H., Jiang F. T., Chen S. (2021). Improving konjac glucomannan-based
aerogels filtration properties by combining aerogel pieces in series
with different pore size distributions. Int.
J. Biol. Macromol..

[51] He L., Ye D. Z., Weng S., Jiang X. C. (2022). A high-strength,
environmentally stable, self-healable, and recyclable starch/PVA organohydrogel
for strain sensor. Eur. Polym. J..

[52] Spagnol C., Rodrigues F. H. A., Pereira A. G. B., Fajardo A. R., Rubira A. F., Muniz E. C. (2012). Superabsorbent hydrogel nanocomposites based on starch-g-poly­(sodium
acrylate) matrix filled with cellulose nanowhiskers. Cellulose.

[53] Zhou C. J., Wu Q. L., Yue Y. Y., Zhang Q. G. (2011). Application of rod-shaped
cellulose nanocrystals in polyacrylamide hydrogels. J. Colloid Interface Sci..

[54] Konieczny J., Loos K. (2018). Facile Esterification
of Degraded and Non-Degraded Starch. Macromol.
Chem. Phys..

[55] Evdokimova O. L., Kusova T. V., Ivanova O. S., Shcherbakov A. B., Yorov K. E., Baranchikov A. E., Agafonov A. V., Ivanov V. K. (2019). Highly
reversible photochromism in composite WO3/nanocellulose films. Cellulose.

[56] Shooto N. D., Wankasi D., Sikhwivhilu L. M., Dikio E. D. (2016). Modified Electro-spun
Polyvinyl Alcohol Nanofibers Used as Super Adsorbing Material for
Lead Ions in Aqueous Solution. J. Residuals
Sci. Technol..

[57] Zhang Y. N., Cui J. Y., Xu S. A. (2015). Effects
of chain structures of corn
starches on starch-based superabsorbent polymers. Starch/Stärke.

[58] Ahmadi-Abhari S., Woortman A. J. J., Hamer R. J., Loos K. (2015). Rheological properties
of wheat starch influenced by amylose-lysophosphatidylcholine complexation
at different gelation phases. Carbohydr. Polym..

[59] Deng C. C., Brooks W. L. A., Abboud K. A., Sumerlin B. S. (2015). Boronic Acid-Based
Hydrogels Undergo Self-Healing at Neutral and Acidic pH. ACS Macro Lett..

[60] Fan X., Geng J., Wang Y., Gu H. (2022). PVA/gelatin/β-CD-based
rapid self-healing supramolecular dual-network conductive hydrogel
as bidirectional strain sensor. Polymer.

[61] Zhao J., Zhao X. Y., Leng L. F., Xu J., Yang X. X., Cui W. X., Zheng J. P., Hu R. F. (2023). High-stretchable,
self-healing, self-adhesive, self-extinguishing, low-temperature tolerant
starch-based gel and its application in stimuli-responsiveness. Carbohydr. Polym..

[62] Zhao L., Ren Z. J., Liu X., Ling Q. J., Li Z. J., Gu H. B. (2021). A Multifunctional,
Self-Healing, Self-Adhesive, and Conductive Sodium
Alginate/Poly­(vinyl alcohol) Composite Hydrogel as a Flexible Strain
Sensor. ACS Appl. Mater. Interfaces.

[63] Tang W., Fu C., Xia L., Lyu P., Li L., Fu Z., Pan H., Zhang C., Xu W. (2022). A flexible and sensitive strain sensor
with three-dimensional reticular structure using biomass Juncus effusus
for monitoring human motions. Chem. Eng. J..

