# Supplementary material for: Self-Healing, Remoldable, and Conductive Starch-Based Dual Reversible Cross-Linking Hydrogels for Strain Sensors
Source: ACS Appl Mater Interfaces. 2025 Jun 22;17(26):38438–50. doi: 10.1021/acsami.5c05168 (PMC12232281; doi:10.1021/acsami.5c05168)
Supplement: Supplementary file 1 [file am5c05168_si_001.pdf]

Supporting Information

Self-Healing, Remoldable, and Conductive Starch-based Dual Reversible Crosslinking Hydrogels for Strain Sensors

*Kai Lu [a, b§], Xiaolong He [c§], Dian Burhani [a, b, d], Jintao Hu [a], Petra Rudolf [c], Dina Maniar [a], Rudy Folkersma [b], Vincent S.D. Voet [b, *], and Katja Loos [a, *]*

[a] *Macromolecular Chemistry and New Polymeric Materials, Zernike Institute for Advanced Materials, University of Groningen, Nijenborgh 3, 9747AG, Groningen, The Netherlands*

[b] *Circular Plastics, Academy Tech & Design, NHL Stenden University of Applied Sciences, Van Schaikweg 94, 7811KL, Emmen, The Netherlands*

[c] *Surfaces and Thin Films, Zernike Institute for Advanced Materials, University of Groningen, Nijenborgh 3, 9747AG Groningen, The Netherlands*

[d] *Research Center for Biomass and Bioproduct, National Research and Innovation Agency, KST Soekarno, Jl. Raya KM.46, Cibinong 16911, Indonesia*

* Email: vincent.voet@nhlstenden.com

* Email: k.u.loos@rug.nl

§ *K.L. and X.L.H. contributed equally to this work*

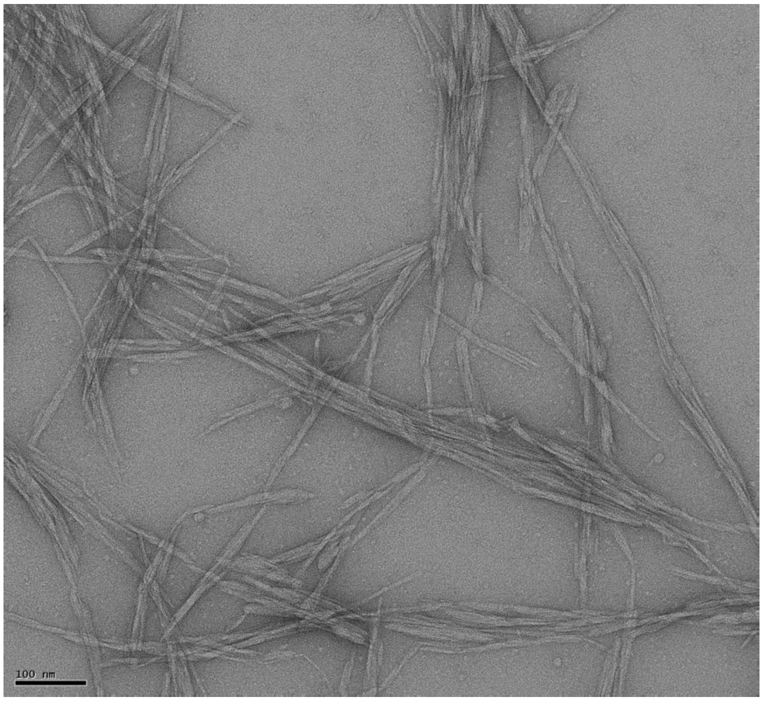

Figure S1. TEM micrograph of CNCs.

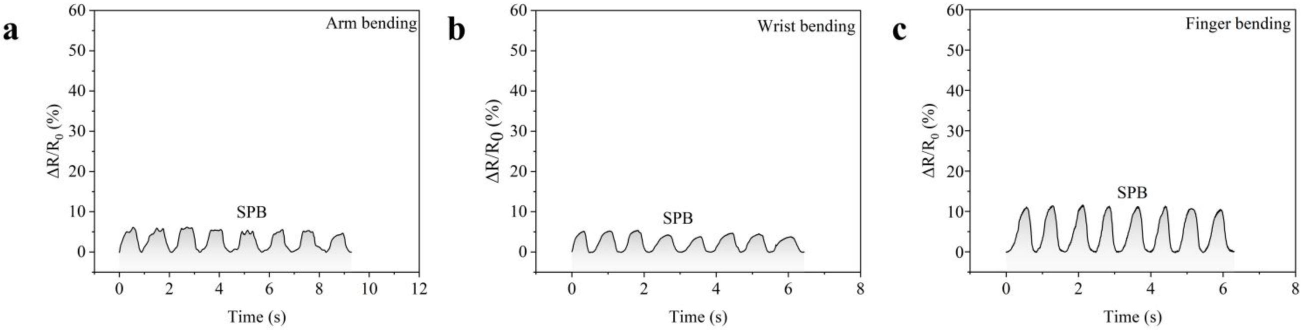

Figure S2. Application demonstration of strain sensors based on the SPB hydrogel. (a) Arm bending; (b) wrist bending; (c) finger bending.
